# VISTA in Soft Tissue Sarcomas: A Perspective for Immunotherapy?

**DOI:** 10.3390/cancers14041006

**Published:** 2022-02-16

**Authors:** Markus Albertsmeier, Annelore Altendorf-Hofmann, Lars H. Lindner, Rolf D. Issels, Eric Kampmann, Hans-Roland Dürr, Martin K. Angele, Frederick Klauschen, Jens Werner, Achim A. Jungbluth, Thomas Knösel

**Affiliations:** 1 Department of General, Visceral and Transplantation Surgery, University Hospital, Ludwig-Maximilians-Universität (LMU) Munich, 81377 Munich, Germany; martin.angele@med.uni-muenchen.de (M.K.A.); jens.werner@med.uni-muenchen.de (J.W.); 2 Department of General, Visceral and Vascular Surgery, Friedrich-Schiller Universität Jena, 07747 Jena, Germany; annelore.altendorf-hofmann@gmx.de; 3 Department of Internal Medicine III, University Hospital, Ludwig-Maximilians-Universität (LMU), 81377 Munich, Germany; lars.lindner@med.uni-muenchen.de (L.H.L.); rolf.issels@med.uni-muenchen.de (R.D.I.); kampmann77@gmail.com (E.K.); 4 Musculoskeletal Oncology, Department of Orthopaedic Surgery, Physical Medicine and Rehabilitation, University Hospital, Ludwig-Maximilians-Universität (LMU) Munich, 81377 Munich, Germany; hans_roland.duerr@med.uni-muenchen.de; 5 Institute of Pathology, Ludwig-Maximilians-Universität (LMU) Munich, 81377 Munich, Germany; frederick.klauschen@med.uni-muenchen.de; 6 Department of Pathology, Memorial Sloan Kettering Cancer Center (MSKCC), New York, NY 10065, USA; jungblua@mskcc.org

**Keywords:** soft tissue sarcoma, immune checkpoint regulator, VISTA, human, systemic therapy

## Abstract

**Simple Summary:**

V domain immunoglobulin suppressor of T cell activation (VISTA) has recently been described as a protein expressed on immune cells and tumour cells and a possible target for immunotherapy. We show for the first time that VISTA is broadly expressed across subtypes of soft tissue sarcoma. We found VISTA related to other immunopathological parameters such as tumour-infiltrating lymphocytes and observed improved survival in patients with non-T-cell-inflamed tumours expressing VISTA. Our research supports the notion of VISTA as a potential target for immunotherapy in soft tissue sarcoma.

**Abstract:**

(1) Background: V domain immunoglobulin suppressor of T cell activation (VISTA) plays a critical role in antitumor immunity and may be a valuable target in cancer immunotherapy. To date, it has never been studied in a large and well-characterised cohort of soft tissue sarcomas (STS). (2) Methods: Using immunohistochemistry, we examined VISTA expression in tumour tissues of 213 high-risk STS. We then analysed whether VISTA was associated with other clinicopathological parameters, including tumour-infiltrating lymphocyte (TIL) counts, programmed death receptor-1 (PD1), programmed death ligand-1 (PDL1), CD3, grading, and long-term survival. (3) Results: We observed VISTA expression in 96 (45%) of 213 specimens with distinct patterns ranging from 26 to 63% for histological subtypes. VISTA was associated with higher grade (G3 vs. G2, *p* = 0.019), higher TIL counts (*p* = 0.033), expression of PD1 (*p* = 0.046), PDL1 (*p* = 0.031), and CD3+ (*p* = 0.023). In patients without CD3^+^ TILs, 10-year survival was higher when VISTA was expressed compared to when there was no VISTA expression (*p* = 0.013). In a multivariate analysis, VISTA expression was independently associated with prolonged survival (*p* = 0.043). (4) Conclusions: VISTA is expressed in different STS subtypes and is associated with increased TILs, PD-1, PD-L1, and CD3 expression. Patients with VISTA^+^ tumours show improved survival. These results may help define future immunotherapeutic approaches in STS.

## 1. Introduction

Malignant soft tissue sarcomas (STS) are a group of more than 100 rare but histopathologically distinct tumours. Originating from mesenchymal tissue, their clinical behaviour ranges from slow growth in grade 1 liposarcoma to local aggressiveness with high recurrence rates in undifferentiated pleomorphic sarcoma or frequent distant metastases in leiomyosarcoma. We regularly apply systemic therapy in high-risk, recurrent, and metastatic patients. Still, the choice of treatment is sometimes difficult due to a paucity of specific recommendations [1,2], and the prognosis of advanced cases remains limited. Following advances in other cancers and the identification of potential targets in STS, different immunotherapeutic approaches have been proposed, including immune checkpoint inhibition, adoptive cell transfer, and tumour vaccinations [3,4]. In clinical trials, however, the efficacy of such strategies was often found to be limited, and novel approaches need to be investigated [5].

V-domain Ig-containing suppressor of T-cell activation (VISTA) is an immune checkpoint gene highly expressed in the environment of malignant tumours that suppresses T-cell activation and induces Foxp3 expression [6]. As a negative checkpoint regulator that shares homology with PD-L1, tumour cells may exploit VISTA to oppose an attack from anti-tumour T-cells. Like PD-1 and PD-L1-directed immunotherapy, VISTA blockade offers a potential immunotherapeutic strategy against cancer. To our knowledge, VISTA has not been described in STS. Therefore, we attempted to analyse the expression of VISTA in a large cohort of mixed STS on the protein level and correlate our results to clinic-pathological parameters and survival in STS patients.

## 2. Materials and Methods

### 2.1. Patients

We identified 213 STS cases from the archives of the Institute of Pathology at Ludwig-Maximilians-Universität (LMU) Munich, Germany. Patients had been diagnosed with high-risk STS (G2/G3 FNCLCC grading, size > 5 cm, deep tumour location) between 1989 and 2012 and received neoadjuvant multimodal treatment at LMU University Hospital and partnering institutions. From the database of the previously published EORTC-STBSG 62961 trial (NCT00003052) [7] and original pathology reports, we retrieved basic clinical data and tumour-related information, such as histological subtype, grading, primary site, size, presence of metastatic disease, and surgical outcome. In addition, we recorded whether chemotherapy, regional hyperthermia, or radiotherapy had been performed as a part of multimodal treatment. We followed patients in outpatient clinics or contacted their general practitioner, updating clinical data until December 2019. The ethics committee at LMU Munich approved this study (protocol code 21-0216).

### 2.2. Histopathology and Tissue Microarray Construction

Whenever possible, we used biopsies that had been taken before the initiation of neoadjuvant treatment from areas of the tumour that appeared dedifferentiated on preoperative cross-sectional imaging (*n* = 165, 77.5%); in 48 patients (22.5%), only tissue samples from surgical specimens were available for this analysis. Two investigators (E.K. and T.K.) reassessed the microscopic findings of the original pathology report, such as tumour type, according to the current WHO classification and degree of differentiation.

For tissue microarray (TMA) assembly, we marked representative tumour areas on H&E-stained slides of formalin-fixed, paraffin-embedded tumour probes, as described previously [8], and took two 0.6 mm punch biopsies from each sample. Samples from tonsils were added as controls. Standard 5 µm TMA sections were prepared for further analysis.

### 2.3. VISTA Immunohistochemistry

We defined VISTA expression on tumour cells as the primary outcome of this study. Following morphological evaluation, we used D1L2G antibodies (Cell Signaling Technologies, Danvers, MA, USA; dilution 1:200) for the immunohistochemical staining of TMAs. We performed all assays on a Leica Bond-3 automated stainer platform (Leica, Buffalo Groves, IL, USA). Antigens were retrieved with heat employing a high-pH buffer (ER2, Leica) before applying the primary antibody. A polymeric secondary antibody kit (Refine, Leica) was used to detect the primary antibody. Two investigators blinded to the clinical data (M.A. and T.K.) evaluated and semi-quantitatively scored immunostaining on a four-tier scale: 0, negative; 1, weak; 2, moderate; and 3, strongly positive. This scale was reduced to a 2-tier system (no expression vs. expression) for statistical analysis. We scored five fields in each sample and selected the higher score for analysis from duplicate probes. Samples with discrepant scores from the two investigators (*n* = 10) were jointly reviewed, reaching a consensus.

### 2.4. TILs, CD3, PD-1 and PD-L1

Recently, TILs, PD-1, and PD-L1 were investigated in this STS cohort [9]. We used those results to explore the relationship between VISTA and the PD-1/PD-L1 pathway in STS. TILs between tumour cells were counted per high-power field (HPF) (400× magnification, field of view 0.237 mm^2^) in H&E-stained TMA slides, as routinely carried out by the pathologist.

As described previously [9], slides were pre-treated with heat and Target Retrieval solution (S1699, Agilent, Santa Clara, CA, USA) before incubation with the monoclonal primary anti-PD-1 mouse antibody (315M; 1:80; Cell Marque, Rocklin, CA, USA) for 60 min at room temperature. The Vectastain Elite ABC HRP Kit (Vector Laboratories, Burlingame, CA, USA) and the chromogen DAB+ (Agilent) were used for detection and Hematoxylin (Vector Laboratories) for counterstaining.

For PD-L1 staining, slides were pre-treated with heat and the Epitope Retrieval Solution pH8 Novocastra (Leica Biosystems, Wetzlar, Germany) before incubation with the monoclonal primary anti-PD-L1 rabbit antibody (E1L3N; 1:50; Cell Signalling Technology) for 60 min at room temperature.

For CD3 staining, a monoclonal antibody raised in rabbit (SP7; 1:150; Zytomed, Berlin, Germany) was employed according to standard procedures. We used the SignalStain Boost IHC Detection Reagent (Cell Signalling Technology) and the chromogen DAB+ (Agilent) for detection.

### 2.5. Statistical Analysis

Data are reported as rates and proportions where applicable. Categorical variables were tested for independence using the Chi-square test; continuous variables were tested with the Kruskal–Wallis test for independent samples or Friedman two-way analysis of variance by ranks, as indicated. Factors associated with VISTA expression were examined in a logistic regression analysis using a stepwise forward approach. We calculated survival from the date when sarcoma was first diagnosed and chose overall survival (death without regard for the cause of death) for estimating prognosis. The Kaplan–Meier method was used to create survival curves, and differences in survival were assessed using the log-rank test. We used the SPSS 26.0 software (IBM, Chicago, IL, USA) for statistical analysis. Statistical significance was defined as a *p*-value < 0.05 for all analyses.

## 3. Results

### 3.1. Patients

The study included 213 patients (108 female, 105 male) with intermediate (G2, *n* = 102) or high-grade (G3, *n* = 111) STS. Their median age at the time of diagnosis was 56 years (range 19 to 79 years). Table 1 reports the clinical parameters related to tumour disease (histological subtype, location, grading, size, presence of metastases) and treatment (surgical outcome, radiotherapy, and regional hyperthermia). All patients received neoadjuvant anthracycline-based systemic therapy. Most patients (*n* = 195, 92%) had primary tumours, while a minority had been referred to us for an early recurrence following ineffective prior treatment. Metastatic disease, if present, had been judged as surgically resectable at initial evaluation. Nearly all patients (*n* = 194, 91%) underwent surgical resection, and about one in five patients received additional radiotherapy.

### 3.2. VISTA Expression in STS and Histopathological Subtypes

**Figure 1:**
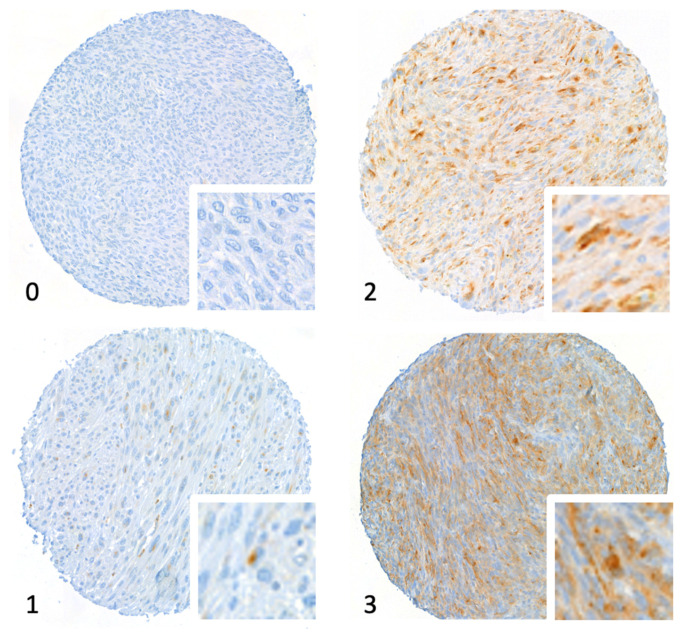
Tissue microarray stained for V domain immunoglobulin suppressor of T cell activation (VISTA). The figure shows core micrographs representative of semiquantitative immunostaining scores: 0, negative; 1, weak; 2, moderate; and 3, strongly positive. Magnification 20×.

We observed VISTA expression in 96 (45%) of 213 samples. Examples of immunohistochemistry staining for VISTA are shown in Figure 1. Dedifferentiated liposarcoma showed lower expression (11/42, 26% positive) compared to other histopathological subtypes (Table 2). In 43 patients for whom tissues samples from both a preoperative biopsy and the surgical specimen were available, we found no difference in VISTA expression before and after neoadjuvant chemotherapy (*p* = 0.251; related-sample Friedman’s two-way analysis of variance on ranks).

### 3.3. VISTA Expression Is Associated with TILs, PD-1, PD-L1 and Grading

The expression of VISTA was related to other immuno-pathological parameters (Table 3). Specifically, VISTA expression was associated with higher TIL counts (*p* = 0.033), increased numbers of CD3^+^ cells (*p* = 0.023), as well as higher expression of PD1 (*p* = 0.046) and PD-L1, (*p* = 0.031). We found no association between VISTA and metastatic disease or tumour location but observed VISTA to be more frequent in patients with higher FNCLCC grade (G3 vs. G2, *p* = 0.019).

We performed a multiple logistic regression analysis of VISTA expression using a stepwise forward inclusion approach with the parameters sex; age; histological subtype; tumour grade; size; surgical margin; metastatic disease; TILs; PD-1, PD-L1, and CD3 expression. Only CD3 expression remained in the final model (OR 3.208 (1.216–8.465), *p* = 0.019). An alternative logistic regression model including most of these parameters is reported in Supplemental Table S1.

### 3.4. VISTA Expression and Survival

The median follow-up duration was 120 months for all patients (IQR 115–120 months). A total of 116 (55%) of the 213 patients died within 10 years of their diagnosis.

We assessed local recurrences and distant metastases after primary therapy only for patients with complete resection of their primary tumour and without distant metastases at diagnosis (*n* = 174). Within this group, 60 patients (35%) developed metastatic disease and 81 patients (47%) developed local recurrence.

**Figure 2:**
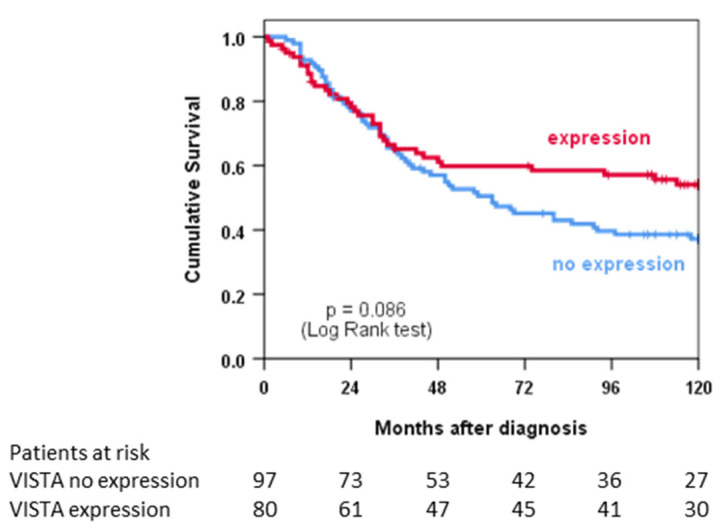
Univariate survival analysis depicted as Kaplan–Meier curves stratified according to VISTA expression.

Figure 2 shows an analysis of overall survival using the Kaplan–Meier method for patients with primary tumours and without metastatic disease (*n* = 177). In univariate analysis, the survival of patients with VISTA^+^ tumours was statistically not significantly different from patients with VISTA^−^ tumours (*p* = 0.086). However, graphical analysis suggests a survival advantage for patients with VISTA^+^ tumours after 48 months. In multivariate analysis, VISTA expression was independently associated with improved survival (*p* = 0.043), while metastatic disease and incomplete resections as well as angiosarcoma and MPNST histotypes were statistically significant risk factors for an unfavourable outcome (Table 4).

**Figure 3:**
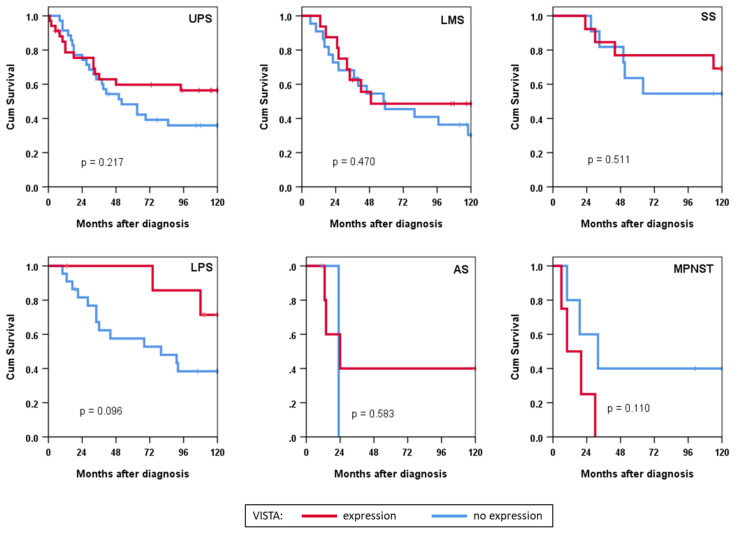
Survival analysis in histotype subgroups depicted as Kaplan–Meier curves stratified according to VISTA expression. UPS: undifferentiated pleomorphic sarcoma; LMS: leiomyosarcoma; SS: synovial sarcoma; LPS: liposarcoma; AS: angiosarcoma; MPNST: malignant peripheral nerve sheath tumour; Cum: cumulative.

In subgroup analyses, we did not observe a statistically significant effect of VISTA expression in histological subtypes (Figure 3). There appeared, however, to be a trend towards improved survival in VISTA-positive patients with dedifferentiated liposarcoma (*p* = 0.096).

**Figure 4:**
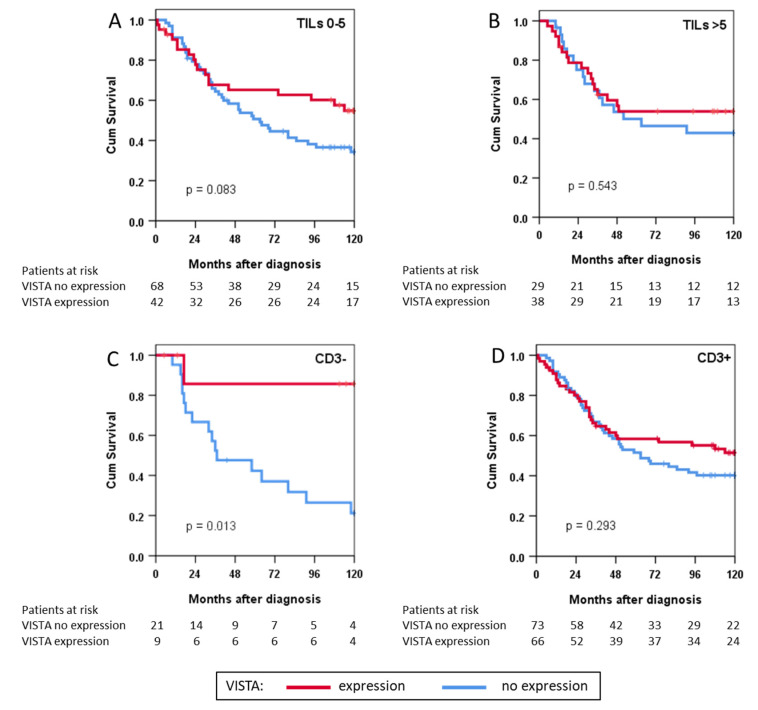
Survival depicted as Kaplan-Meier curves in immunophenotype subgroups of patients with either (**A**) low or (**B**) high tumour-infiltrating lymphocyte (TIL) counts and with (**C**) negative or (**D**) positive CD3 expression; Cum: cumulative.

Finally, we analysed survival for the subgroups of patients with low TILs vs. high TILs and those with CD3^−^ tumours vs. CD3^+^ tumours (Figure 4). We found no statistically significant association of survival with VISTA expression in patients with high or low TILs but observed better survival in patients with VISTA^+^CD3^−^ tumours compared to VISTA^+^CD3^+^ patients (*p* = 0.013).

## 4. Discussion

This study shows that VISTA is frequently expressed across different histological subtypes of STS and that VISTA expression may be associated with improved survival. To the best of our knowledge, this is the first analysis of VISTA on the protein level in a large and well-characterised cohort of STS patients. A strength of this study cohort is the quality and length of patient follow-up. In one study based on the pan-cancer TCGA dataset, sarcomas were among those tumours with the highest VISTA expression [10]. Therefore, protein-level confirmation and the analysis of prognostic relevance in immunologically defined subgroups are valuable contributions to the study of immune checkpoint regulators.

Our study has some limitations. First, the low number of patients per histological subgroup requires the confirmation of the prognostic relevance of VISTA expression in a larger cohort. Second, tumour heterogeneity has been shown to confound measurements of PD-L1 expression in lung cancer [11,12] and may play a role in our analysis of VISTA accordingly, as STS are characterised by substantial intratumoral heterogeneity with distinguishable areas of differentiation [13] co-existing in one tumour. We addressed this problem by taking biopsies from dedifferentiated areas whenever possible. Finally, tissue samples in 22.5% of patients were available only from the surgical specimen after neoadjuvant treatment. Preoperative chemotherapy and regional hyperthermia may have confounded our results [14], although we found no difference in VISTA expression before and after surgery.

VISTA appears to play both suppressive and stimulatory roles in tumour immunology with varying effects and clinical relevance in different tumour types [15]. In an analysis of sarcoma patients from the TCGA database, VISTA expression was associated with prolonged survival [15]. Although the mechanisms leading to this diverse behaviour have only partly been understood, it appears that the tissue (tumour vs. immune cells) and the precise cell type that predominantly expresses VISTA does play a role. In this regard, some studies have described VISTA as an immune checkpoint receptor primarily expressed on TILs and myeloid cells, leading to a suppression of T cell activation, proliferation, and cytokine production. In pancreatic cancer, for example, VISTA appears to be predominantly expressed on CD86^+^ macrophages, and the expression of inhibitory immune checkpoint genes (VISTA and others combined) was associated with shorter survival [16].

Other studies show that VISTA is overexpressed in tumour tissues, suggesting that it acts as a co-stimulatory molecule inhibiting tumour proliferation and progression—e.g., in ovarian cancer [17], oesophageal adenocarcinoma [18], gastric cancer [19], and hepatocellular carcinoma [20]. Unlike these studies, where VISTA was associated with favourable clinical features, we found that VISTA was more frequently expressed in FNCLCC G3 STS but unrelated to metastatic disease. Specifically, VISTA expressed by ovarian cancer cells has been shown to suppress T-cell functions, thus limiting the tumour-directed activity of CD8^+^ TILs and potentially promoting tumour growth [21]. In contrast to this mechanism of action yet in line with previous results [15,17,22], we found VISTA expression on sarcoma cells to be associated with the expression of CD3 (univariate and in a logistic regression model) as well as the presence of TILs and the expression of PD-1 and PD-L1 (univariate), suggesting that VISTA may play a role in the inflamed tumour microenvironment.

In this context, our finding that survival was improved in patients with CD3^−^VISTA^+^ tumours should be interpreted with caution, as this exploratory analysis was not planned. Nonetheless, our results suggest a T-cell-independent influence of VISTA on the tumour microenvironment. Hypothetically, the absence of T-cells directed against the tumour, which VISTA would regulate, might allow VISTA’s assumed direct anti-tumour effects to outweigh cell-mediated tumour growth (VISTA^+^ in Figure 4C,D). However, as the relevant biological aspects concerning its molecular interactions remain unclear, a better understanding of its mechanisms and functions is necessary to establish VISTA as a biomarker for immunotherapy [20,23], especially in STS.

The overexpression of VISTA on tumour cells and the possible survival benefit for patients with CD3^−^VISTA^+^ tumours support VISTA as a target for immunotherapy in STS. Moreover, it has been demonstrated that VISTA is upregulated following PD-1/PD-L1 blockade in various tumours [19,22,24,25,26]. In this regard, the overlapping expression of both checkpoint regulators may open a therapeutic opportunity for tumours that have developed resistance to PD-1/PD-L1 blockade.

Patient selection for immune checkpoint inhibitor therapy is not straightforward due to the complex pathways involved. The experience gained in recent years with melanoma, non-small cell lung cancer, and metastatic colorectal cancer patients points to the existence of additional risk factors that need to be incorporated [27], such as tumour-mutational burden or TILs in melanoma. We propose the investigation of VISTA as a predictive marker in STS patients treated with immune checkpoint inhibitor therapy.

## 5. Conclusions

In summary, the results of our study indicate that VISTA is expressed in various sarcoma subgroups. VISTA expression on tumour cells is associated with elevated TILs; the expression of PD-1, PD-L1, and CD3; and higher FNCLCC grading. Patients with VISTA^+^ tumours appear to survive longer than VISTA^−^ patients. The analysis of VISTA expression may guide future immunotherapeutic approaches to STS.

## Figures and Tables

**Table 1:** Patients’ baseline characteristics. UPS: undifferentiated pleomorphic sarcoma; MPNST: malignant peripheral nerve sheath tumour.

**Variation**		***n***	**%**
**Total**		213	100
**Sex**	Male	105	49
	Female	108	51
**Histological** **subtype**	UPS	77	36
	Leiomyosarcoma	47	22
	Synovial sarcoma	27	13
	Dedifferentiated liposarcoma	34	16
	Myxoid liposarcoma	9	4
	Angiosarcoma	8	4
	MPNST	11	5
**Location**	Extremities	71	33
	Retroperitoneal	45	21
	Abdominal/visceral	35	17
	Trunk	55	26
	Other	7	3
**Grading**	Intermediate (G2)	102	48
	High (G3)	111	52
**Size**	<50 mm	17	8
	50–79 mm	48	22
	80–120 mm	62	29
	>120 mm	63	30
	Missing	23	11
**Primary tumour/ ** **recurrence**	Primary tumour	195	92
	Recurrence	18	8
**Metastatic disease**	M0	195	92
	M1	18	8
**Surgical ** **margins**	R0/R1	186	87
	R2 or no resection	27	13
**Radiotherapy**	Done	168	79
	Not done	45	21
**Regional ** **hyperthermia**	Done	164	77
	Not done	49	23
**Chemotherapy**	Neoadjuvant/perioperative	213	100
	Adjuvant only	0	0

**Table 2:** V domain immunoglobulin suppressor of T cell activation (VISTA) expression and TILs for different histologic subtypes: The table shows the number and percentage of patients with different VISTA expression scores (0 to 3) and positive expression (score 1 to 3) and high tumour-infiltrating lymphocyte (TIL) infiltration (≥6 TILs per HPF). UPS: undifferentiated pleomorphic sarcoma; DDLPS: dedifferentiated liposarcoma; MPNST: malignant peripheral nerve sheath tumour.

**Variation**	**VISTA Expression**										**TILs**		
**Histological**	**0**		**1**		**2**		**3**		**Positive**		**Positive**		**Total**
**Subtype**	*n*	%	*n*	%	*n*	%	*n*	%	*n*	%	*n*	%	*n*
**UPS**	38	49%	31	40%	7	9%	1	1%	39	51%	37	48%	77
**Leiomyosarcoma**	25	53%	21	45%	1	2%	0	0%	22	47%	16	34%	47
**Synovial sarcoma**	14	52%	13	48%	0	0%	0	0%	13	48%	3	11%	27
**Liposarcoma**	32	74%	10	23%	1	2%	0	0%	11	26%	17	40%	43
**DDLPS**	25	74%	8	24%	1	3%	0	0%	9	26%	15	44%	34
**Myxoid**	7	78%	2	22%	0	0%	0	0%	2	22%	2	22%	9
**Angiosarcoma**	3	38%	4	50%	1	13%	0	0%	5	63%	4	50%	8
**MPNST**	5	45%	6	55%	0	0%	0	0%	6	55%	3	27%	11
**Total**	117	55%	85	40%	10	5%	1	0%	96	45%	80	38%	213

**Table 3:** Association between V domain immunoglobulin suppressor of T cell activation (VISTA) expression and clinicopathological parameter grading: TILs, tumour-infiltrating lymphocytes. The Chi-square test was used to test for the independence of variables.

**Variation**		**VISTA**					
		**Total**	**Expression**		**No Expression**		***p***
**TILs**	0–5	133	52	39%	81	61%	0.033
	≥ 6	80	44	55%	36	45%	
	Total	213	96	45%	117	55%	
**CD3**	No expression	34	9	26%	25	74%	0.023
	Expression	170	82	48%	88	52%	
	Total	204	91	45%	113	55%	
**PD-1**	0–3	145	58	40%	87	60%	0.046
	≥ 4	61	34	56%	27	44%	
	Total	206	92	45%	114	55%	
**PD-L1**	No expression	171	73	43%	98	57%	0.031
	Expression	31	20	65%	11	35%	
	Total	202	93	46%	109	54%	
**Metastasis**	M0	195	85	44%	110	56%	0.215
	M1	18	11	61%	7	39%	
	Total	213	96	45%	117	55%	
**Location**	Extremities	73	37	51%	36	49%	0.249
	Non-extremities	140	59	42%	81	58%	
	Total	213	96	45%	117	55%	
**Grade**	Intermediate	102	37	36%	65	64%	0.019
	High	111	59	53%	52	47%	
	Total	213	96	45%	117	55%	

**Table 4:** Multivariate survival analysis using a Cox regression model. TILs, tumour-infiltrating lymphocytes; UPS: undifferentiated pleomorphic sarcoma; MPNST: malignant peripheral nerve sheath tumour.

Variation		HR (95% CI)	*p*-Value
**Age**	Increase by 1 year	1.015 (0.999–1.031)	0.059
**Sex**	Female (vs. male)	0.800 (0.525–1.218)	0.298
**VISTA**	No expression (vs. expression)	1.014 (1.014–2.363)	0.043
**TILs**	≥6 (vs. 0–5)	1.125 (0.726–1.743)	0.597
**CD3**	No expression (vs. expression)	0.982 (0.525–1.838)	0.956
**PD-1**	≥4 (vs. 0–3)	1.104 (0.696–1.750)	0.674
**PD-L1**	No expression (vs. expression)	0.890 (0.488–1.622)	0.703
**Size**	≥8 cm (vs. <8 cm)	1.358 (0.866–2.129)	0.183
**Metastasis**	M1 (vs. M0)	1.986 (1.008–3.912)	0.047
**Grade**	Grade 1/2 (vs. Grade 3)	1.446 (0.937–2.231)	0.095
**Surgical outcome**	R2/not resected (vs. R0/1)	5.936 (3.390–10.393)	<0.001
**Histotype**	UPS (Reference)		0.075
	Leiomyosarcoma	1.246 (0.727–2.135)	0.424
	Synovial sarcoma	1.012 (0.474–2.159)	0.975
	Liposarcoma	0.800 (0.450–1.423)	0.447
	Angiosarcoma	3.992 (1.099–14.497)	0.035
	MPNST	2.461 (1.010–5.995)	0.047

## Data Availability

The data presented in this study are available on specific request from the corresponding author. The data are not publicly available for reasons of data protection and data economy.

## Supplementary Materials

The following supporting information can be downloaded at: https://www.mdpi.com/article/10.3390/cancers14041006/s1. Table S1: Multiple logistic regression model of clinicopathological parameters.
