# Supplementary material for: VISTA in Soft Tissue Sarcomas: A Perspective for Immunotherapy?
Source: Cancers (Basel). 2022 Feb 16;14(4):1006. doi: 10.3390/cancers14041006 (PMC8870227; doi:10.3390/cancers14041006)
Supplement: Supplementary file 1 [file cancers-14-01006-s001.zip › cancers-1582431-supplementary.pdf]

Supplemental Table S1: Multiple logistic regression model of clinicopathological parameters. TILs: Tumour-infiltrating lymphocytes; UPS: Undifferentiated pleomorphic sarcoma; MPNST: Malignant peripheral nerve sheath tumour.

		**OR (95% CI)**	***p*-value**
**Age**	increase by 1 year	1.027 (1.002–1.054)	0.037
**Sex**	female (vs male)	1.944 (0.979–3.864)	0.058
**TILs**	≥ 6 (vs 0–5)	1.436 (0.701–2.943)	0.322
**CD3**	no expression (vs expression)	2.275 (0.762–6.792)	0.141
**PD-1**	≥ 4 (vs 0–3)	1.824 (0.821–4.051)	0.140
**PD-L1**	no expression (vs expression)	2.065 (0.769–5.545)	0.150
**Size**	≥ 8 cm (vs < 8 cm)	1.433 (0.689–2.977)	0.335
**Metastasis**	M1 (vs M0)	1.985 (0.532–7.403)	0.307
**Grade**	Grade 1/2 (vs Grade 3)	0.625 (0.303–1.288)	0.202
**Surgical outcome**	R2/not resected (vs R0/1)	0.368 (0.127–1.064)	0.065
**Histotpye**	UPS (Reference)	0.226	
	Leiomyosarcoma	0.506 (0.109–2.346)	0.384
	Synovial sarcoma	0.748 (0.153–3.651)	0.720
	Liposarcoma	1.013 (0.198–5.190)	0.987
	Angiosarcoma	0.233 (0.047–1.160)	0.075
	MPNST	0.269 (0.020–3.597)	0.321
	Constant	0.034	
